# Role of Quzhou Fructus Aurantii Extract in Preventing and Treating Acute Lung Injury and Inflammation

**DOI:** 10.1038/s41598-018-20083-z

**Published:** 2018-01-26

**Authors:** Lili Li, Sheng Zhang, Yanfei Xin, Junying Sun, Feng Xie, Lin Yang, Zhiqin Chen, Hao Chen, Fang Liu, Yaoxian Xuan, Zhenqiang You

**Affiliations:** 1 Collaborative Innovation Center of Yangtze River Delta Region Green Pharmaceuticals, Zhejiang University of Technology, Hangzhou, Zhejiang, China; 2 State Key Laboratory of Safety Evaluation for New Drugs, Zhejiang Academy of Medical Sciences, Hangzhou, Zhejiang, China; 3 Institute of Animal Health, Guangdong Academy of Agricultural Sciences, Guangzhou, Guangdong, China

## Abstract

Quzhou Fructus Aurantii (QFA) is an authentic herb of local varieties in Zhejiang, China, which is usually used to treat gastrointestinal illnesses, but its effects on respiratory inflammation have not been reported yet. In our study, the anti-inflammatory activity of QFA extract (QFAE) was evaluated on copper sulfate pentahydrate (CuSO_4_·5H_2_O)-induced transgenic neutrophil fluorescent zebrafish model. QFAE showed a significant effect of anti-inflammation in CuSO_4_·5H_2_O-induced zebrafish by reducing the neutrophil number in the inflammatory site. We investigated the anti-inflammatory activity of QFAE on lipopolysaccharide (LPS)-induced acute lung injury (ALI) mice models and RAW 264.7 cells. QFAE had an anti-inflammatory effect on reducing total cells, neutrophils, and macrophages in BALF and attenuated alveolus collapse, neutrophils infiltration, lung W/D ratio, myeloperoxidase (MPO) protein expression and other pulmonary histological changes in lung tissues, as well as hematological changes. Levels of pro-inflammatory cytokines, including TNF, IL-6, IFN-γ, MCP-1, and IL-12p70, were decreased, whereas anti-inflammatory cytokine IL-10 was increased after treatment with QFAE both *in vivo* and *in vitro*. In summary, our results suggested that QFAE had apparent anti-inflammatory effects on CuSO_4_·5H_2_O-induced zebrafish, LPS-induced ALI mice, and RAW 264.7 cells. Furthermore, QFAE may be a therapeutic drug to treat ALI/ARDS and other respiratory inflammations.

## Introduction

Respiratory inflammation is a feature of many respiratory diseases, including acute lung injury (ALI) and its more severe manifestation, acute respiratory distress syndrome (ARDS), which are closely bound up with sepsis^1,2^, pneumonia^3^, and trauma^4^. Environmental and genetic factors also lead to the susceptibility and severity of ALI/ARDS^5^. ALI/ARDS is characterized by a sudden onset respiratory failure following acute hypoxaemia, pulmonary infiltrates, pulmonary hypertension, pulmonary edema, decreased pulmonary compliance, neutrophil accumulation, and gas-exchange abnormality, and is responsible for high rates of morbidity and mortality worldwide^6–8^. According to epidemiology, ALI/ARDS can appear in patients of all ages and lacks proper therapeutic medicine^7,9,10^. Thus, a therapeutic drug to treat ALI is urgently needed.

Quzhou Fructus Aurantii (QFA) is recorded in the “Zhejiang Traditional Chinese Medicine Processing Norms (2015),” which makes QFA have an official and legitimate medicinal identity and become an authentic herb. QFA is a dried unripe fruit of Rutaceae Citrus changshan-huyou Y.B. Chang, which is a natural hybrid of grapefruit and other citruses in Changshan County, Quzhou City, Zhejiang Province, and has been cultivated for several hundred years. QFA is usually used to treat gastrointestinal illnesses in traditional Chinese medicine^11^. However, the effect of QFA on respiratory inflammation has not been reported. Previous studies have shown that QFA has no significant difference in chemical composition from Fructus Aurantii (FA)^12^, which confirmed that the QFA derived from Changshan is similar to the FA in Chinese Pharmacopoeia based on characters and compositions. Previous studies have shown that FA has high antioxidant activity and anti-inflammatory effects and promotes gastrointestinal peristalsis and cardiovascular protection^13–16^. In the present study, we evaluated whether QFA extract (QFAE) could alleviate acute lung injury in LPS-induced ALI mice model or attenuate acute inflammation in CuSO_4_·5H_2_O-induced zebrafish model and LPS-induced RAW 264.7 cell model.

## Methods

### Ethics statement

The study design and protocols were approved by the Ethical Committee of Zhejiang Academy of Medical Sciences. All experiments were carried out in accordance with relevant guidelines and regulations. All individuals signed an informed consent to participate in the study.

### Preparation of QFAE

Exactly 0.2 kg dried QFA at 2.5 months of fruit period was crushed into coarse particles, added with 6 times its volume of deionized water, soaked for 1 h, and then distilled. Subsequently, 100 mL distillate was collected for the standby application. Afterward, the dregs were heated and refluxed twice. The first time was added 10 times its volume of deionized water to reflux for 1 h, and the second time was added 5 times its volume of deionized water to reflux for 0.5 h. Filtrates were combined after two filtrations and concentrated under reduced pressure to 100 mL. Finally, 100 mL filtrate was combined with the previous 100 mL distillate to obtain 200 mL of 1 g/mL of the QFAE and stored at 4 °C.

### HPLC analysis of naringin in QFAE

**Figure 1:**
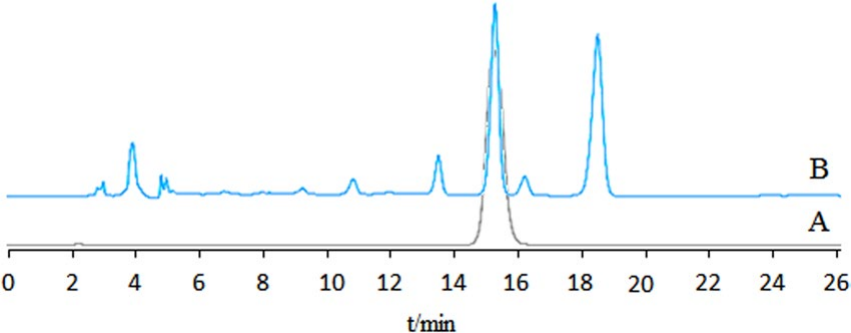
HPLC chromatogram obtained at 283 nm from (**A**) standard product of naringin, (**B**) QFAE.

HPLC (Agilent Technologies) was used for the determination of naringin in QFAE. Naringin was separated on a Gemini 5μ C18 110 A column (250 mm × 4.60 mm, 5 micro) (Phenomenex) with acetonitrile-0.5% acetic acid (volume ratio of 22:78) as the mobile phase. The UV detection wavelength was set at 283 nm; flow rate was 1.0 mL/min; column temperature was set at 35 °C; and injection volume was 20 μL. The standard product of naringin was purchased from the National Institutes for Food and Drug Control. Retention time was 15.470 min (Fig. 1) and the content of naringin in QFAE was 11.12 mg/mL.

### Animals and treatments

A total of 90 male specific pathogen-free (SPF) ICR mice weighing approximately 18–22 g were purchased from Shanghai Slack Laboratory Animal Co., Ltd. The mice were housed in microisolator cages and given ad libitum access to food and water. All mice were groomed in the barrier system with clean environment at 20 °C to 25 °C under 50–60% humidity and with 12 h bright and 12 h dark lighting cycle. Animal experiments were conducted in laboratories that passed the authentication of the Association for Assessment and Accreditation of Laboratory Animal Care (AAALAC).

The mice were randomly divided into 6 groups, with 15 mice each: a blank control group, a drug control (QFAE 25 g/kg) group, an LPS group, and QFAE (2.5, 7.5, and 25 g/kg) + LPS group. On days 1–2 of the experiment, QFAE was administered intragastrically to the drug control group and QFAE + LPS group. Mice in the blank control group and LPS group received the same treatments of saline. On day 3, all mice (excepted for blank control and drug control groups) were given light anesthesia via intraperitoneal administration with 0.5% pentobarbital sodium and intranasal administration with 20 μg of LPS (Sigma-Aldrich) in 50 μL of PBS to induce acute lung injury. Blank control and drug control groups received 50 µL of PBS. After 4 h, all mice were continued to be given intragastric administration with the original drug and original dose. On day 4, the mice were sacrificed and we collected samples of blood, bronchoalveolar lavage fluid (BALF), and lung tissues.

Transgenic neutrophil fluorescent zebrafish naturally mating in pairs were at 3 days post fertilization (dpf). Zebrafish were housed in 28 °C fish water (water quality: 200 mg instant sea salt per 1 L of reverse osmosis water, with a conductivity of 480 to 510 μS/cm; pH of 6.9 to 7.2; and hardness of 53.7 to 71.6 mg/L CaCO_3_). Animal experiments were conducted in laboratories that passed the authentication of AAALAC.

Maximum tolerated concentration of transgenic neutrophil zebrafish was determined in the pre-experiment. A total of 180 zebrafish were randomly housed in a 6-well plate with 30 zebrafish each: a blank control group, a model control group (CuSO_4_·5H_2_O, Xilong Chemical. Co., Ltd.), a positive control group [indomethacin 25 μg/mL, Aladdin Reagent (Shanghai). Co., Ltd., China], and QFAE (222, 667, and 2000 µg/mL) groups, and the capacity of each well was 3 mL. After each group was treated for 1 h, except for the blank control group, the rest of the groups were given CuSO_4_·5H_2_O to induce zebrafish inflammation models. After 2 h of treatment, 10 zebrafish were randomly selected from each group and photographed under fluorescence microscope. Number of neutrophils in the inflammatory sites was analyzed. Ratio of inflammatory inhibition was calculated based on the number of neutrophils. The calculation formula was given as follows:

$$\frac{{\rm{Model}}\,{\rm{Control}}\,\mathrm{Group}-\mathrm{Treatment}\,{\rm{Group}}}{{\rm{Model}}\,{\rm{Control}}\,\mathrm{Group}-\mathrm{Blank}\,{\rm{Control}}\,{\rm{Group}}}\times 100 \% $$

The treatment group represented for positive control (indomethacin 25 μg/mL) group and QFAE (222, 667, and 2000 µg/mL) groups.

### Blood collection and analysis

Blood samples of mice were taken from the retrobulbar venous plexus and divided into heparinized tubes and centrifuge tubes. The former were shaken well for hematological examination, and the latter were centrifuged at 5000 rpm for 10 min at 4 °C to collect serum and then stored at −80 °C for later analysis of cytokine levels following the manufacturer’s instructions by Mouse Inflammation Kit (BD^TM^ Cytometric Array) using the flow cytometer (BD FACSCalibur^TM^ Flow Cytometer).

### BALF acquisition and analysis

The lungs of mice were lavaged thrice with 0.8 mL saline, and the BALF was centrifuged at 2000 rpm for 5 min at 4 °C. The cells were harvested for cell counting. Then, parts of the cells were resuspended in DMEM medium, plated onto a 96-well plate for cell classification, and observed with an optical microscope. Another part of the cells was used for Western blot analysis to detect the expression of myeloperoxidase (MPO). The cell-free supernatant was stored at −80 °C for later analysis of cytokine levels, which was performed by Mouse Inflammation Kit using the flow cytometer.

### Lung wet-to-dry weight ratio (W/D ratio)

Lung W/D ratio indicates the degree of pulmonary edema. Lungs were excised to obtain their wet weight (W). Subsequently, the lungs were dried in an incubator at 80 °C for 48 h and weighed to obtain the dry weight (D). The W/D ratio was calculated to assess pulmonary edema.

### H&E staining and histopathological examination

Lung tissues were fixed in 10% formaldehyde solution and embedded in paraffin. Lung sections were stained with hematoxylin and eosin (H&E). Neutrophils and alveolar state were observed with an optical microscope.

### Immunohistochemistry analysis

Lung tissues were fixed in 10% formaldehyde solution, embedded in paraffin, cut into 5 μm sections, and then dewaxed, hydrated, and retrieved in citrate buffer. Then, endogenous peroxidase activity and nonspecific binding were blocked. Subsequently, sections were incubated overnight at 4 °C in a humid chamber with myeloperoxidase (MPO) monoclonal antibody (1:1000, abcam). On the second day, the sections were washed in PBS and incubated with horseradish peroxidase-labeled goat anti-rabbit IgG (1:2000, Hangzhou Baoke Biotechnology. Co., Ltd., China) at 37 °C for 30 min. After washing with PBS, the sections were dyed in DAB (3, 3′-diaminobenzidine), redyed in hematoxylin, differentiated in 1% hydrochloric-alcohol solution, dehydrated, and sealed. Presence of buffy or brown diaminobenzidine precipitates is indicative of positive reactivity, which was observed with an optical microscope.

### Western blot

Cells from BALF were lysed in RIPA buffer and centrifuged at 12,000 g for 10 min at 4 °C. Total protein concentration was determined by BCA Protein Assay Kit. Then, the samples were separated by 10% sodium dodecyl sulfate-polyacrylamide gel electrophoresis (SDS-PAGE) and electrophoretically transferred onto polyvinylidene fluoride membranes. Membranes were blocked with 5% nonfat milk for 1 h at room temperature, followed by incubation with primary antibody against MPO overnight at 4 °C and horseradish peroxidase-conjugated second antibody for 1 h at room temperature. Immunoreactive proteins were visualized with an enhanced chemiluminescence (ECL) Western blot detection system. Western blot quantifications were analyzed by Image J software.

### Cell culture and treatment

RAW 264.7 (a murine macrophage cell line) was purchased from Shanghai Gefan Biotechnology. Co., Ltd., China. Cells were cultured in Dulbecco’s modified Eagle’s medium (DMEM) supplemented with 10% heat-inactivated fetal bovine serum, 100 IU/mL penicillin, and 100 μg/mL streptomycin at 37 °C in a humidified atmosphere with 5% CO_2_.

RAW 264.7 cells were plated onto 6-well plates (1 × 10^5^ cells/well) and incubated in the presence of QFAE (0.2, 1, and 5 mg/mL) for 1 h before LPS stimulation (1 μg/mL). The blank control and drug control groups were set at the same time. Cell-free supernatants were collected after 24 h and stored at −80 °C for later analysis of cytokine levels by Mouse Inflammation Kit using the flow cytometer.

### Statistical analysis

Results were reported as mean ± standard error of mean (SEM) and were analyzed using SPSS 22.0. One-way ANOVA was conducted to compare data among more than two groups. *P* < 0.05 was considered statistically significant.

## Results

### Effect of QFAE on neutrophil number in inflammatory site from CuSO_4_·5H_2_O-induced transgenic neutrophil fluorescent zebrafish model

**Figure 2:**
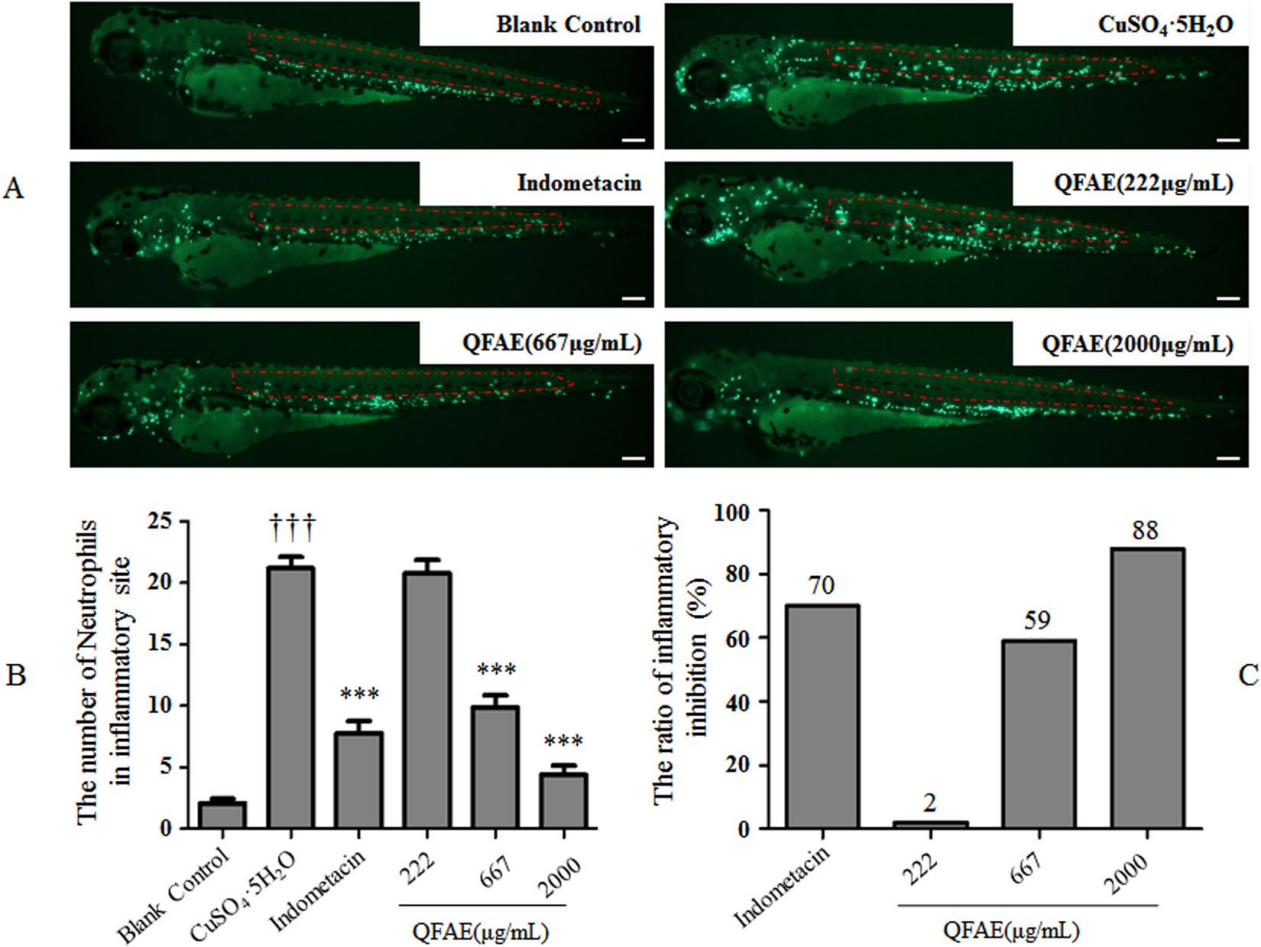
Effect of QFAE on inflammatory sites in CuSO_4_·5H_2_O-induced transgenic neutrophil fluorescent zebrafish. (**A**) Typical photos of neutrophils in inflammatory site of CuSO_4_·5H_2_O-induced transgenic neutrophil fluorescent zebrafish model after QFAE treatment. The green fluorescent spots are neutrophils, and the red area is a quantitative inflammatory site. The green fluorescent spots in the red area are the neutrophils of inflammatory site. Scale bar, 100 μm. (**B**) Effect of QFAE on neutrophil number in inflammatory site from CuSO_4_·5H_2_O-induced zebrafish model. ***P < 0.001 compared with that of model control group (CuSO_4_·5H_2_O), ^†††^*P* < 0.001 compared with the blank control group. (**C**) Effect of QFAE on ratio of inflammatory inhibition in CuSO_4_·5H_2_O-induced zebrafish model.

We found that the neutrophil number in inflammatory site in the model control group (CuSO_4_·5H_2_O) was significantly higher than that in the blank control group, which indicated the success of the CuSO_4_·5H_2_O-induced zebrafish model. The positive control (indomethacin 25 μg/mL) group indicated that indomethacin significantly inhibited the neutrophil number in the inflammatory site compared with that of the model control group. Meanwhile, QFAE (222, 667, and 2000 µg/mL) treatment groups showed a dose-dependent effect on reducing the neutrophil number in the inflammatory site. We found that the anti-inflammatory effect of QFAE (2000 µg/mL) treatment group was better than that of the positive control (indomethacin 25 μg/mL) group (Fig. 2A,B). Furthermore, the ratio of inflammatory inhibition in QFAE (2000 µg/mL) treatment group (88%) was notably higher than that in the positive control (indomethacin 25 μg/mL) group (70%), which indicated that QFAE had a striking anti-inflammatory effect (Fig. 2C).

### Effect of QFAE on cytokine levels in LPS-induced RAW 264.7

**Figure 3:**
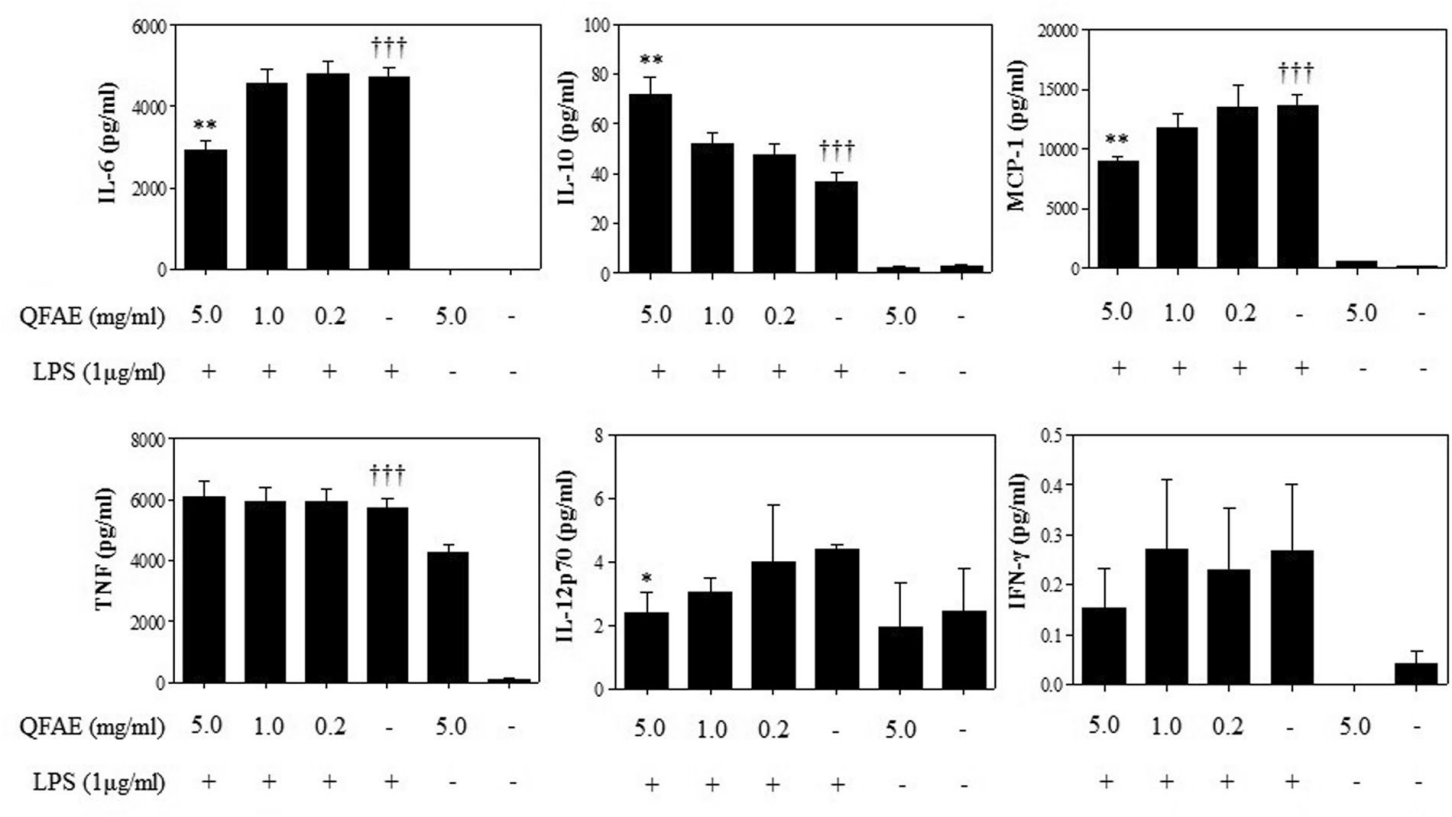
Effect of QFAE on cytokine levels in LPS-induced RAW 264.7. Data are presented as the means ± SEM, *P < 0.05 and **P < 0.01 compared with the LPS group, ^†††^*P* < 0.001 compared with the blank control group.

We determined the cytokines, including IL-6, IL-10, IL-12p70, TNF, IFN-γ, and MCP-1, using Mouse Inflammation Kit using the flow cytometer. IL-6, IL-12p70, TNF, IFN-γ, and MCP-1 are pro-inflammatory cytokines, and IL-10 is an anti-inflammatory cytokine. As demonstrated in Fig. 3, the levels of pro-inflammatory cytokines and anti-inflammatory cytokine IL-10 were clearly increased in LPS-induced RAW 264.7 in the LPS group compared with that in the blank control group. In our study, we discovered that the treatment with QFAE (5 mg/mL) resulted in a clear decrease on the levels of IL-6, IFN-γ, MCP-1, and IL-12p70 in LPS-induced RAW 264.7. However, QFAE did not significantly suppress the levels of TNF in LPS-induced RAW 264.7. In addition, the level of anti-inflammatory cytokine IL-10 was apparently enhanced after treatment with QFAE (5 mg/mL) compared with that of the LPS group.

### Effect of QFAE on inflammatory cells in BALF from LPS-induced mice

**Figure 4:**
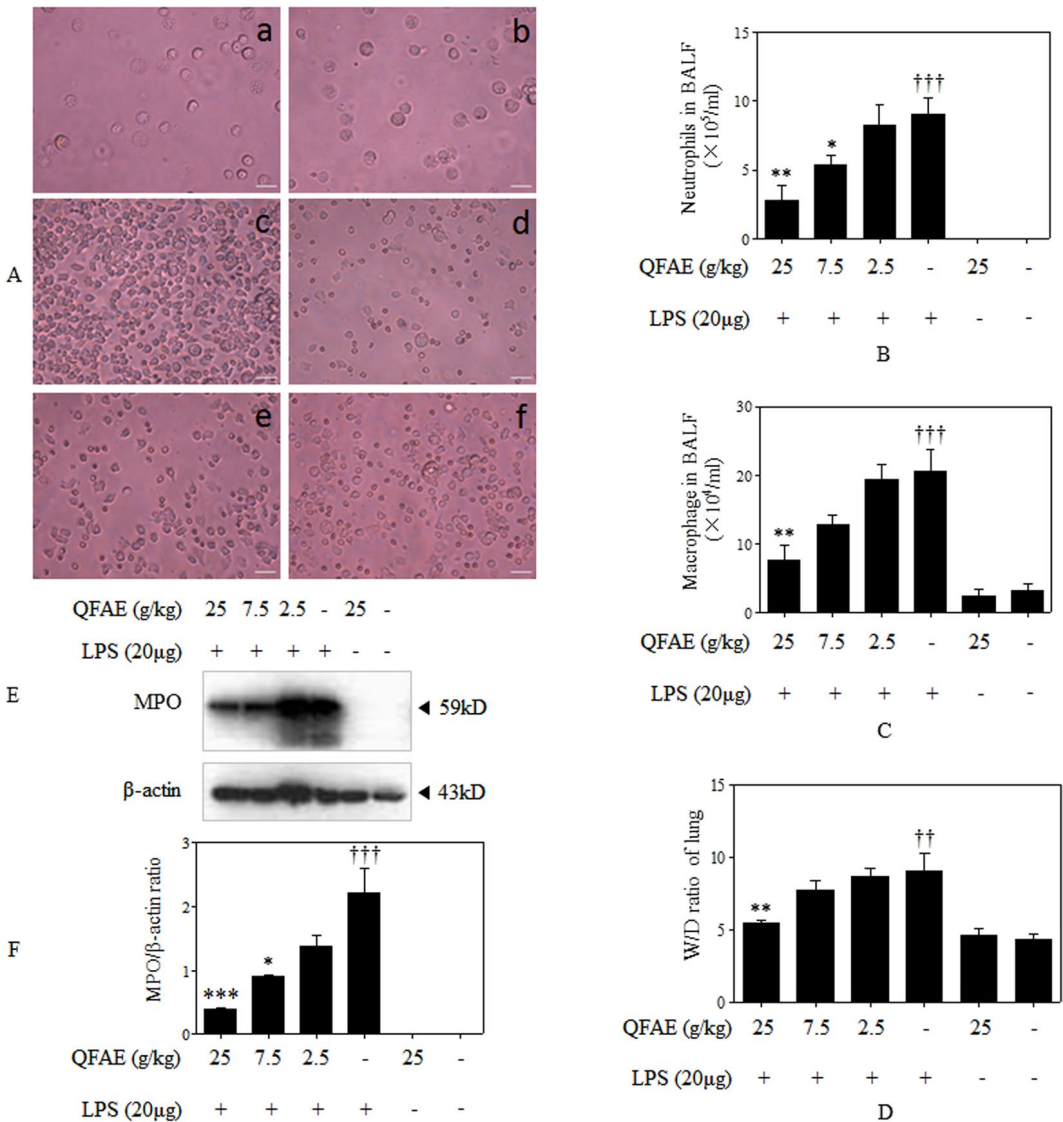
Effect of QFAE on pulmonary inflammation in ALI mice induced by LPS. (**A**) Effect of QFAE on number of inflammatory cells in BALF from LPS-induced mice (100× ). (a) Blank control group, (b) Drug control group, (c) LPS group, (d) QFAE (25 g/kg) + LPS groups, (e) QFAE (7.5 g/kg) + LPS groups, (f) QFAE (2.5 g/kg) + LPS groups. Scale bar, 100 μm. (**B**) Effect of QFAE on neutrophils in BALF. (**C**) Effect of QFAE on macrophages in BALF. (**D**) Effect of QFAE on lung W/D ratio. (**E**) Effect of QFAE on expression of MPO in cells from BALF by Western blot. (**F**) Protein level of MPO was normalized to the level of β-actin, the loading control. Data are presented as the means ± SEM, *P < 0.05, **P < 0.01 and ***P < 0.001 compared with the LPS group, ^††^*P* < 0.01 and ^†††^*P* < 0.001 compared with the blank control group.

To identify the anti-inflammation property of QFAE, cell counting and cell classification in BALF were performed in our study. Neutrophils and macrophages are typical inflammatory cells. After the stimulation of LPS, total cells, neutrophils and macrophages were obviously increased in BALF. However, QFAE treatment groups had a dose-dependent effect on reducing total cells, neutrophils and macrophages in BALF. In addition, QFAE (25 g/kg) treatment group observably reduced total cells, neutrophils and macrophages compared with those in the LPS group (Fig. 4A–C).

### Effect of QFAE on lung W/D ratio in LPS-induced mice

Lung W/D ratio indicates the degree of pulmonary edema. We found that LPS notably increased the W/D ratio, which indicated severe pneumonedema. However, QFAE treatment groups reduced the W/D ratio compared with that of the LPS group. QFAE (25 g/kg) treatment group was significantly decreased compared with that of the LPS group (Fig. 4D).

### Effect of QFAE on expression of MPO protein in cells from BALF by Western blot

MPO, which can indirectly reflect the amount of neutrophil and degree of inflammation, is exported by neutrophils during the inflammatory process^17,18^. In our study, Western blot was used to measure the MPO protein expression levels in BALF cells. MPO protein expression was markedly higher in the LPS group than in the blank control and drug control groups. We noted a significant decrease in the expression of MPO protein in the BALF cells of mice treated with QFAE, especially in QFAE (25 and 7.5 g/kg) treatment groups (Fig. 4E,F).

### Effect of QFAE on histopathological changes in lung tissues from LPS-induced mice

**Figure 5:**
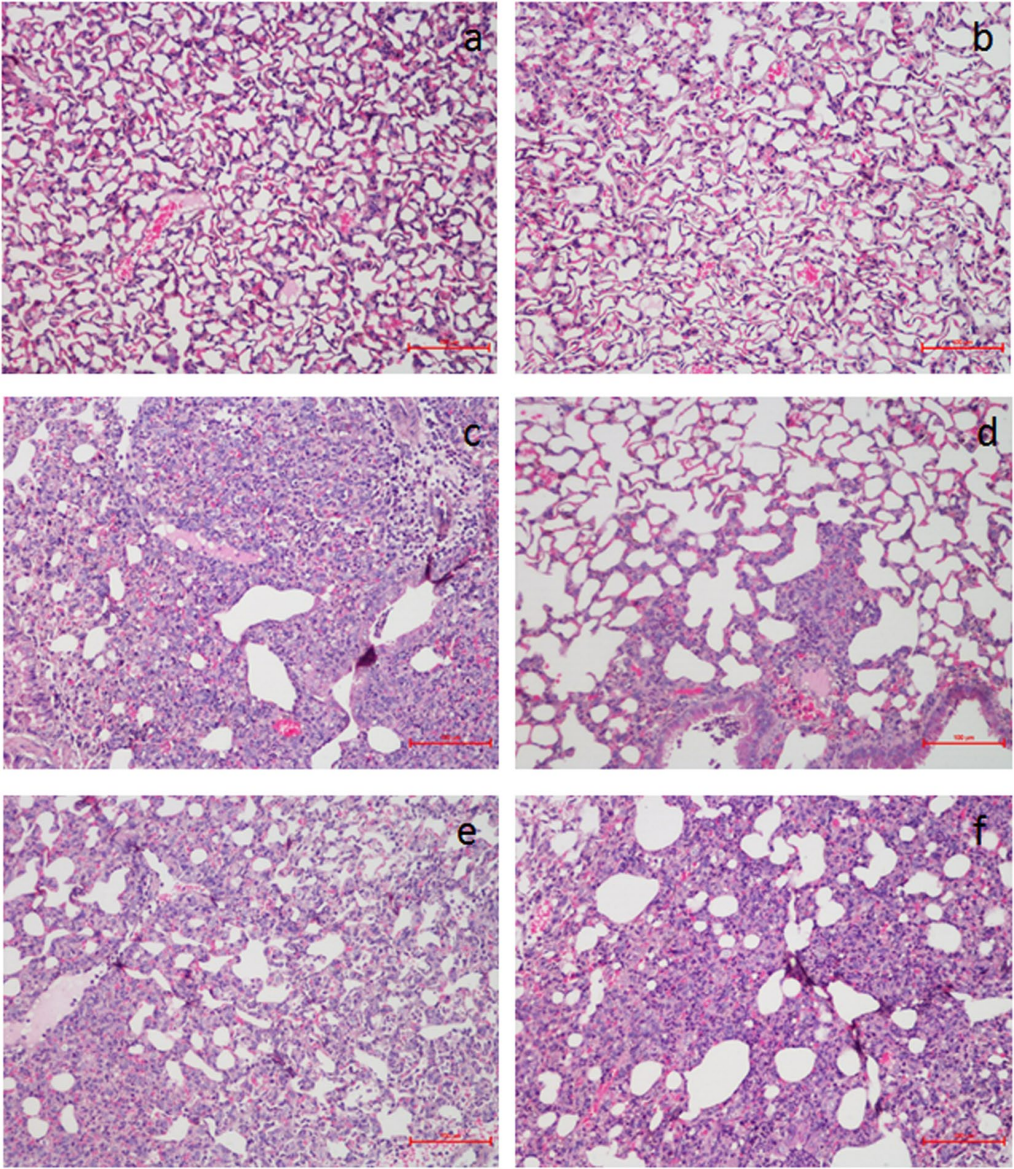
Effect of QFAE on histopathological changes in lung tissues from LPS-induced mice (200×). (**a**) Blank control group, (**b**) Drug control group, (**c**) LPS group, (**d**) QFAE (25 g/kg) + LPS groups, (**e**) QFAE (7.5 g/kg) + LPS groups, (**f**) QFAE (2.5 g/kg) + LPS groups. Scale bar, 100 μm.

H&E staining was used to determine the degree of damage in LPS-induced ALI. Blank control and drug control groups showed normal pulmonary structures with no histopathological changes (Fig. 5a,b). In the LPS group, the lungs were markedly damaged with severe alveolar hemorrhage, alveolar collapse, and distinct neutrophil infiltration, which indicated the success of the ALI model (Fig. 5c). Nevertheless, QFAE treatment groups alleviated the above histopathological changes in a dose-dependent manner (Fig. 5d–f).

### Effect of QFAE on immunohistochemical changes in lung tissues from LPS-induced mice

**Figure 6:**
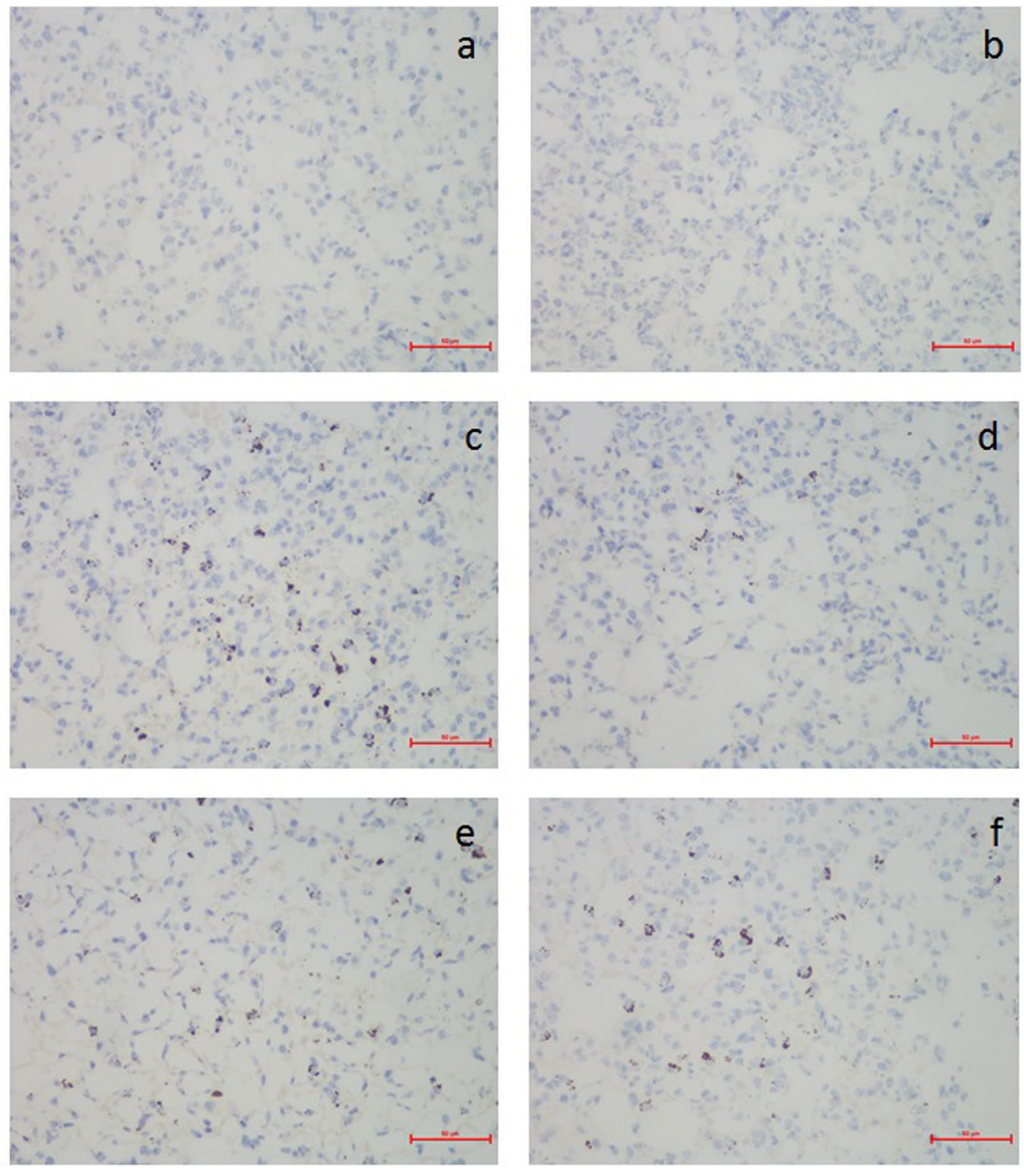
Effect of QFAE on immunohistochemical changes in lung tissues from LPS-induced mice (400×). (**a**) Blank control group, (**b**) Drug control group, (**c**) LPS group, (**d**) QFAE (25 g/kg) + LPS groups, (**e**) QFAE (7.5 g/kg) + LPS groups, (**f**) QFAE (2.5 g/kg) + LPS groups. Scale bar, 50 μm.

As shown in Fig. 6a,b, few specific expressions of MPO protein were observed in the blank control and drug control groups. However, the expression of MPO protein in the LPS group (Fig. 6c) was dramatically higher than that in the blank control and drug control groups. QFAE treatment groups significantly attenuated the expression of MPO protein compared with that in the LPS group (Fig. 6d–f). Meanwhile, QFAE (25 g/kg) treatment group demonstrated that the expression of MPO protein was weak.

### Effect of QFAE on cytokine levels in cell-free supernatant from BALF and serum

**Figure 7:**
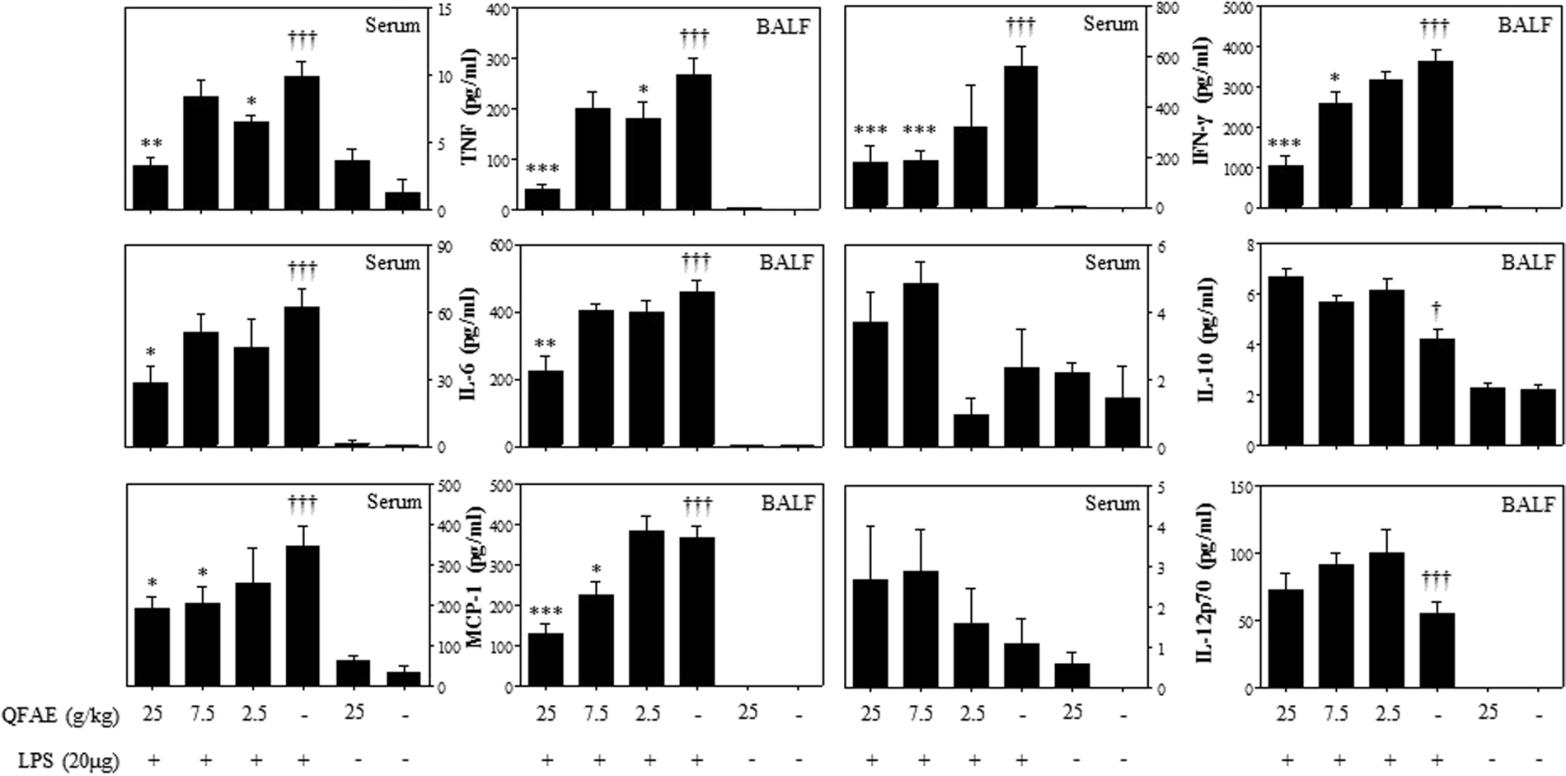
Effect of QFAE on cytokine levels in cell-free supernatant from BALF and serum. Data are presented as the means ± SEM, *P < 0.05, **P < 0.01 and ***P < 0.001 compared with the LPS group, ^†††^*P* < 0.001 compared with the blank control group.

As illustrated in Fig. 7, the levels of pro-inflammatory cytokines in BALF and serum in the LPS group were markedly increased compared with that of the blank control group. Treatment with QFAE (25 g/kg) led to a significant decrease in the levels of IL-6, TNF, IFN-γ, and MCP-1 in the BALF and serum. However, QFAE did not significantly inhibit the levels of IL-12p70 in BALF and serum. Furthermore, the levels of anti-inflammatory cytokine IL-10 in BALF and serum were increased after treatment with QFAE in ALI mice compared with that of the LPS group.

### Effect of QFAE on hematological changes in LPS-induced mice

Hematological examination indicated that neutrophil (NEUT) was significantly increased in the LPS group in LPS-induced mice compared with that in the blank control group. Simultaneously, NEUT was apparently decreased in the QFAE (25 and 7.5 g/kg) treatment groups compared with that in the LPS group. Lymphocyte (LYMPH) was apparently inhibited in the LPS group in LPS-induced mice compared with that in the blank control group. Meanwhile, LYMPH was distinctly increased in the QFAE (7.5 g/kg) treatment group compared with that in the LPS group. However, QFAE (2.5 g/kg) treatment group did not show anti-inflammatory effect on hematological changes, and QFAE had no significant effect on white blood cell (WBC), platelet (PLT), and monocyte (MONO), as shown in Table 1.

**Table 1:** Effect of QFAE on hematological changes in LPS-induced mice.

Groups	WBC (×10^3^/μl)	PLT (×10^3^/μl)	NEUT (×10^3^/μl)	LYMPH (×10^3^/μl)	MONO (×10^3^/μl)
Blank Control	2.61 ± 0.31	1327.23 ± 54.15	0.34 ± 0.04	2.29 ± 0.32	0.05 ± 0.01
QFAE (25 g/kg)	2.74 ± 0.18	1316.54 ± 139.61	0.46 ± 0.13	2.74 ± 0.45	0.04 ± 0.01
LPS	2.66 ± 0.31	1177.33 ± 411.16	1.37 ± 0.26^†††^	0.84 ± 0.46^†^	0.02 ± 0.01
QFAE (25 g/kg) + LPS	3.20 ± 0.19	1542.67 ± 392.02	0.58 ± 0.31**	1.06 ± 0.56	0.03 ± 0.01
QFAE (7.5 g/kg) + LPS	2.91 ± 0.31	1096.00 ± 204.16	0.96 ± 0.25*	1.58 ± 0.54*	0.03 ± 0.01
QFAE (2.5 g/kg) + LPS	2.18 ± 0.37	1110.08 ± 103.27	1.32 ± 0.21	0.96 ± 0.13	0.02 ± 0.01

Data are presented as the means ± SEM, *P < 0.05 and **P < 0.01 compared with the LPS group, ^†^p < 0.05 and ^†††^p < 0.001 compared with the blank control group. Note: white blood cell (WBC), platelet (PLT), neutrophil (NEUT), lymphocyte (LYMPH), monocyte (MONO).

## Discussion

QFA is a unique local citrus variety in Changshan County, Quzhou City, Zhejiang Province, and its traits and utility are not different from FA in the China Pharmacopoeia; moreover, QFA has a long history in Quzhou City as a folk conventional medicine. In this study, we aimed to study the anti-inflammatory activities of QFAE *in vitro* and *in vivo*. Previous studies have shown that FA contains naringin, hesperidin, synephrine, narirutin, neohesperidin, didymin, nobiletin, tangeretin, auraptene, neoeriocitrin, poncirin, and other substances^19–21^, which have anti-inflammatory effects because of its components, such as naringin^22–24^, hesperidin^23,24^, synephrine^25^, narirutin^26^, neohesperidin^27^, and nobiletin^28^. In our experiment, we detected the content of naringin in QFAE, which was used as a criterion for the quality control and quantification of QFAE.

Zebrafish as a novel animal model has received focus from scientists. The main characteristics and advantages of zebrafish are as follows: small, easy feeding, rapid development, short sexual maturity, strong fecundity, embryonic development *in vitro*, easy to observe and operate, improved embryo and genetic manipulation technology, abundant breeds and resources, real-time imaging, and annotation of genomic sequences^29–31^. Because of the transparency of the larval zebrafish, the accessibility of transgenic fluorescent reporter lines, and the maintenance of cellular components of the innate immune system, immunological studies can be conducted on the behavior of infiltrating cells in living animals. Transgene-marked fluorescent zebrafish neutrophils play an effective role in the study of acute inflammation^32,33^. Commonly used inducers are LPS, Poly IC, silica nanoparticles, zinc and copper^34–36^. Copper is a metal capable of inducing inflammatory injury and cellular migration to the hair cells of lateral line of zebrafish and can easily imitate antioxidant and pro-inflammatory characteristics. Transient exposure of larval zebrafish in copper is a frequently used method to establish acute inflammation models^37–39^. In our study, we used CuSO_4_·5H_2_O to induce transgenic neutrophil fluorescent zebrafish modeling acute inflammation, and the accumulation of neutrophils was significantly increased. After treating with QFAE, the number of neutrophils was clearly reduced, which showed the excellent anti-inflammatory effects of QFAE.

Deserved to be mentioned, previous literature indicated that FA has a certain therapeutic effect on cardiovascular disease^16,40,41^ and gastrointestinal disorders^42–44^. Therefore, we conducted related research tests to QFAE using the zebrafish model. QFAE had an antihypertensive effect on egg yolk powder-hyperlipidemia zebrafish model (data not shown). Moreover, QFAE promoted intestinal peristalsis on Nile red-labeled zebrafish model (data not shown).

Regarding *in vitro* tests, previous studies demonstrated that LPS induces macrophages to release plenty of pro-inflammatory cytokines, including TNF, IL-6, IFN-γ, MCP-1, IL-12p70 and anti-inflammatory cytokine IL-10^45–47^. These inflammatory immunoregulatory molecules have crucial roles in inflammatory diseases. To study the effect of QFAE on cytokine levels *in vitro*, we detected TNF, IL-6, IFN-γ, MCP-1, IL-12p70 and IL-10 levels in LPS-induced RAW 264.7 cells, and QFAE was found to significantly inhibit the levels of pro-inflammatory cytokines, including IL-6, IFN-γ, MCP-1 and IL-12p70. Meanwhile, the levels of anti-inflammatory cytokine IL-6 clearly increased in a dose-dependent manner. These results showed that QFAE has *in vitro* anti-inflammatory effect.

ALI/ARDS is a critical illness syndrome consisting of dyspnea, acute hypoxemic respiratory failure, bilateral pulmonary infiltrates, and pulmonary edema and has a high incidence^48,49^. The pathophysiology of ALI/ARDS is complicated and includes a complex array of molecular, cellular, and physiological mechanisms^50^. Progress in medical research related to ALI/ARDS relies on the continuous development of corresponding biomedicine, and research with animal models of ALI/ARDS is essential. Common ALI animal models are summarized as follows: (1) Adult pigs, dogs and sheep often use bronchoalveolar lavage (BAL) to induce lung injury, which is mainly used for the study of pulmonary surfactant replacement therapy and the lack of pulmonary surfactant in ALI model^51,52^. (2) The oleic acid (OA) model is often used to replicate lung injury caused by lipid embolization in clinical practice and is suitable for various mammals^53,54^. (3) Endotoxin model. Endotoxin is the outer membrane of Gram-negative bacteria, contains LPS as its main pathogenic substance, frequently used to induce ALI/ARDS, and consists of an endotoxin, an oligosaccharide, and a polysaccharide. LPS-induced animal models emphasize methods to research the mechanisms of various illnesses and supply valuable information on the findings of novel biomarkers and drug targets^55,56^. In our study, we administered LPS intranasally to establish an ALI animal model, and the result indicated that LPS successfully resulted in alveolar hemorrhage, alveolar collapse, neutrophil infiltration, lung edema, and other pulmonary histological changes in the ALI mice model. Besides, the levels of pro-inflammatory cytokines were increased, whereas anti-inflammatory cytokine IL-10 was decreased in the ALI mice model. QFAE markedly attenuated LPS-induced histopathological changes in lung tissues, obviously reduced the levels of pro-inflammatory cytokines, and increased the levels of anti-inflammatory cytokine IL-10 in BALF and serum. The results proved that QFAE has remarkable anti-inflammatory function on ALI mice.

MPO is a distinguished enzyme in the innate defense released by neutrophils, macrophages and monocytes during phagocytosis into the extracellular environment, and MPO also takes part in various biological effects^17,57^. MPO is characterized by pro-oxidative and pro-inflammatory properties that can be a feasible marker for a series of inflammatory diseases, including acute coronary syndromes (ACS)^58^, atherosclerosis^59^, acute lung inflammation^60^, and acute intestinal inflammation^18,61^. In our study, we used western blot to detect the expression of MPO in the cells of BALF from LPS-induced ALI mice. The expression of MPO sharply increased after the stimulation of LPS in LPS groups, which indicated serious inflammation in the ALI model, as well as the success of the ALI model. However, a significant reduction on the expression of MPO was found in mice treated with QFAE. Similar results appeared in immunohistochemistry assay. Both western blot and immunohistochemistry assay testified the favorable anti-inflammatory effect of QFAE.

In our pre-experiment, an assay of maximum administration dosage (MAD) of QFAE on ICR mice was conducted. The results revealed that MAD was 100 g/kg, and there was no adverse reaction on mice. In our study, 25 g/kg was chosen as the highest dose. Furthermore, the mice were given QFAE treatment for 9 days in our pre-experiment. However, the concentrations of neutrophil and lymphocyte in hematological examination were significantly higher than those of the mice in the blank control group. These results indicated that the administration cycle of QFAE needs to be adjusted, thus we shortened 9 days to 3 days. The mechanisms that cause these phenomena are not clear at the moment. In the later stage, we will conduct safety evaluation of QFAE and investigate its toxic effect on various organs.

In conclusion, our study revealed that QFAE was able to alleviate LPS-stimulated inflammatory responses in RAW 264.7 cells on cytokine levels, which could decrease the levels of pro-inflammatory cytokines and increase the level of anti-inflammatory cytokine IL-10. Besides, QFAE protected against LPS-induced ALI in mice, which alleviated alveolar collapse, neutrophils infiltration, lung W/D ratio, MPO protein expression, and other pulmonary histological changes in lung tissues, as well as hematological changes and inflammatory cytokine levels. Furthermore, QFAE moderated CuSO_4_·5H_2_O-activated inflammatory effects in transgenic neutrophil fluorescent zebrafish model, which reduced the neutrophil number in inflammatory site. These findings of our study suggested that QFAE has an excellent anti-inflammatory activity both *in vitro* and *in vivo* and may serve as a potential medicine to ALI/ARDS and even other respiratory inflammation cases. Our study is a pharmacodynamic experiment and we did not conduct an in-depth research of anti-inflammatory ingredients and pathways of QFAE. Further experiments are required for exploring the detailed ingredients and mechanism of QFAE to lay the foundation for future clinical therapy.
